# An Antimicrobial Peptide Regulates Tumor-Associated Macrophage Trafficking via the Chemokine Receptor CCR2, a Model for Tumorigenesis

**DOI:** 10.1371/journal.pone.0010993

**Published:** 2010-06-08

**Authors:** Ge Jin, Hameem I. Kawsar, Stanley A. Hirsch, Chun Zeng, Xun Jia, Zhimin Feng, Santosh K. Ghosh, Qing Yin Zheng, Aimin Zhou, Thomas M. McIntyre, Aaron Weinberg

**Affiliations:** 1 Department of Biological Sciences, Case Western Reserve University School of Dental Medicine, Cleveland, Ohio, United States of America; 2 Department of Oral Pathology, Case Western Reserve University School of Dental Medicine, Cleveland, Ohio, United States of America; 3 Department of Chemistry, Cleveland State University, Cleveland, Ohio, United States of America; 4 Department of Otolaryngology-Head and Neck Surgery, University Hospitals of Cleveland, Case Western Reserve University School of Medicine, Cleveland, Ohio, United States of America; 5 Case Comprehensive Cancer Center, Cleveland, Ohio, United States of America; 6 Department of Cell Biology, Lerner Research Institute, Cleveland Clinic College of Medicine of Case Western Reserve University, Cleveland, Ohio, United States of America; University of Toronto, Canada

## Abstract

**Background:**

Tumor-associated macrophages (TAMs) constitute a significant part of infiltrating inflammatory cells that are frequently correlated with progression and poor prognosis of a variety of cancers. Tumor cell-produced human β-defensin-3 (hBD-3) has been associated with TAM trafficking in oral cancer; however, its involvement in tumor-related inflammatory processes remains largely unknown.

**Methodology:**

The relationship between hBD-3, monocyte chemoattractant protein-1 (MCP-1), TAMs, and CCR2 was examined using immunofluorescence microscopy in normal and oral carcinoma *in situ* biopsy specimens. The ability of hBD-3 to chemoattract host macrophages *in vivo* using a nude mouse model and analysis of hBD-3 on monocytic cell migration *in vitro*, applying a cross-desensitization strategy of CCR2 and its pharmacological inhibitor (RS102895), respectively, was also carried out.

**Conclusions/Findings:**

MCP-1, the most frequently expressed tumor cell-associated chemokine, was not produced by tumor cells nor correlated with the recruitment of macrophages in oral carcinoma *in situ* lesions. However, hBD-3 was associated with macrophage recruitment in these lesions and hBD-3-expressing tumorigenic cells induced massive tumor infiltration of host macrophages in nude mice. HBD-3 stimulated the expression of tumor-promoting cytokines, including interleukin-1α (IL-1α), IL-6, IL-8, CCL18, and tumor necrosis factor-α (TNF-α) in macrophages derived from human peripheral blood monocytes. Monocytic cell migration in response to hBD-3 was inhibited by cross-desensitization with MCP-1 and the specific CCR2 inhibitor, RS102895, suggesting that CCR2 mediates monocyte/macrophage migration in response to hBD-3. Collectively, these results indicate that hBD-3 utilizes CCR2 to regulate monocyte/macrophage trafficking and may act as a tumor cell-produced chemoattractant to recruit TAMs. This novel mechanism is the first evidence of an hBD molecule orchestrating an *in vivo* outcome and demonstrates the importance of the innate immune system in the development of tumors.

## Introduction

Collaborative interactions of tumor cells with leukocyte infiltrates in the tumor microenvironment can significantly influence tumor development and progression [1], [2], [3], [4]. Macrophages residing in the tumor site, collectively termed tumor-associated macrophages (TAMs), often constitute a major part of infiltrating leukocytes and represent a significant component of cancer-associated inflammatory environment [5]. Clinical studies have shown that tumor infiltration of macrophages is associated with progression and poor prognosis in more than 80% of cancers, including cancers of breast, prostate, bladder, cervix, and head and neck [6], [7]. Experimental studies using mouse models confirm that genetic and chemical ablation of macrophages leads to an inhibition of tumor progression and reduced rate of metastasis [8], [9], [10], [11]. Tumor-produced factors, including a variety of cytokines, activate TAMs to stimulate tumor cell proliferation, migration, angiogenesis, metastasis [5], [8], [12]. TAMs derive from circulating monocytes that are selectively recruited to the tumor site by chemotactic factors locally produced by tumor and stromal cells. Experimental and clinical studies have shown that monocyte chemoattractant peptide-1 (MCP-1), also known as chemokine (C-C motif) ligand 2 (CCL2), is perhaps the chemokine most frequently expressed by tumor cells and is correlated with recruitment of host macrophages to the tumor site in a variety of human tumors, such as sarcomas, gliomas, melanomas, cancers of the breast, cervix, and ovary [6], [7]. Other chemokines, such as CCL5, CCL7, CCL8, and CXCL12, as well as tumor cell-produced growth factors, such as vascular endothelial growth factor (VEGF), transforming growth factor-β (TGF-β), and fibroblast growth factor (FGF), are also described as chemotactic for monocytes/macrophages during tumor development [7], [13]. These chemotactic factors, based on clinical and experimental studies, are summarized in Table 1. Although lipopolysaccharides (LPS) treatment induces production of MCP-1 and CCL20, as well as IL-6 in cultured oral cancer cell lines *in vitro*, the contribution of these cytokines in recruiting monocyte-lineage cells in oral carcinoma *in situ* and invasive OSCC *in vivo* is still unknown [14]. In head and neck squamous cell carcinoma (HNSCC), infiltration of macrophages into and around cancer tissues is significantly correlated with tumor size, aggressiveness, invasion, and poor prognosis [15], [16], [17]. However, chemotactic molecules that participate in the recruitment of inflammatory cells in HNSCC are still largely undetermined. In oral squamous cell carcinoma (OSCC), the expression of MCP-1 and CCL5 have detected in scattered non-neoplastic inflammatory cells, while only a few of MCP-1 expressing tumor cells have been found in less than 40% of cases studied [18]. Therefore, the collective findings to date suggest that other tumor cell-produced factors may chemoattract immune cells. Our recent findings have shown that tumor cells overwhelmingly produce human β-defensin-3 (hBD-3), but not MCP-1, in the oral CIS lesion [19]. Kesting et al. have confirmed our observations by reporting overexpression of hBD-3 in oral cancer tissues using paired cancerous and noncancerous specimens derived from 46 patients [20]. HBD-3, therefore, may play an important role in the development and progression of oral cancer.

**Table 1:** Chemotactic molecules involved in inflammatory cell trafficking.

Chemoattractants	Receptors	Associated tumors	References
MCP-1/CCL2	CCR2	sarcoma, gliomas, melanomas, lung, breast, cervix, ovary, colon.	[6], [7], [72], [73]
CCL3	CCR5	lung, breast, hepatocellular carcinoma, multiple myeloma, chronic lymphocytic leukemia, colon.	[73], [74], [75], [76], [77]
CCL4	CCR5	chronic lymphocytic leukemia, colon.	[75], [76]
CCL5/RANTES	CCR1, CCR5	breast, melanoma, cervix	[78], [79], [80], [81], [82]
CXCL8/IL-8	IL8R	melanoma, lung	[72]
CXCL12/SDF1-α	CXCR4	breast, ovarian	[83], [84]
M-CSF	CSF1R	sarcoma	[85], [86]
GM-CSF	CSF2R	breast and others	[87], [88], [89]
VEGFA	VEGFR1	lung and others	[90], [91], [92]
TGFβ	TGFR	breast, lung	[91], [93], [94]
C5a	C5AR1	cervix	[95], [96], [97]

RANTES, Regulated upon Activation Normal T-cell Expression and presumably Secreted; C5a, complement 5a; M-CSF, Macrophage Colony-Stimulating Factor; GM-CSF, Granulocyte-Macrophage Colony Stimulating Factor.

Human β-defensins (hBDs) are small cationic peptides originally identified from the plasma of patients with renal disease (hBD-1) and from psoriatic skin lesions (hBD-2 and hBD-3) as antimicrobial agents of innate immunity [21], [22], [23]. It has now been reported that hBDs display a variety of biological activities, particularly their participation in “cross-talking” with the adaptive immune system [24]. HBD-3 has been shown to antagonize the HIV co-receptor CXC chemokine receptor 4 (CXCR4) and to activate professional antigen-presenting cells (APCs) via heterodimerized TLR1 and 2 [25], [26]. The chemokine receptor CCR6 has been shown to mediate migration of memory T cells and iDCs in response to hBD-1 and -2 [27]. However, the membrane receptor(s) that mediates hBD-3-induced monocytic cell migration has yet to be identified, as CCR6 is not expressed on this cell type [19], [28], [29]. Although the *in vitro* information being gathered to date implies that hBDs have the capacity to immunoregulate the adaptive immune system, whether they participate in actual regulation of immune responses *in vivo*; i.e., inflammatory processes in tumor pathogenesis, is largely unknown. We previously demonstrated that hBD-3 over-expression by tumor cells in oral CIS lesions is correlated with recruitment and infiltration of macrophages to the tumor site [19]. In addition, hBD-3 chemoattracts monocytic THP-1 cells *in vitro*, suggesting a possible connection between hBD-3 expression and TAM trafficking [19].

In the present study, we demonstrate that tumor cells do not express MCP-1 in oral CIS biopsies. Moreover, xenograft tumors generated by tumorigenic cells that overexpress hBD-3 show massive host macrophage infiltration and enhanced tumorigenicity when compared with those formed by parent cells. In addition, hBD-3 induced monocytic cell migration is blocked by cross-desensitization with MCP-1 and by the treatment of cells with the specific CCR2 inhibitor, RS102895, respectively. Collectively, these results support our hypothesis that hBD-3 functions as a chemoattractant to recruit macrophages and that CCR2 plays a central role in mediating monocyte/macrophage migration in response to hBD-3.

## Results

### Association of the hBD-3-rich tumor environment with intratumoral accumulation of CCR2 expressing cells

**Figure 1:**
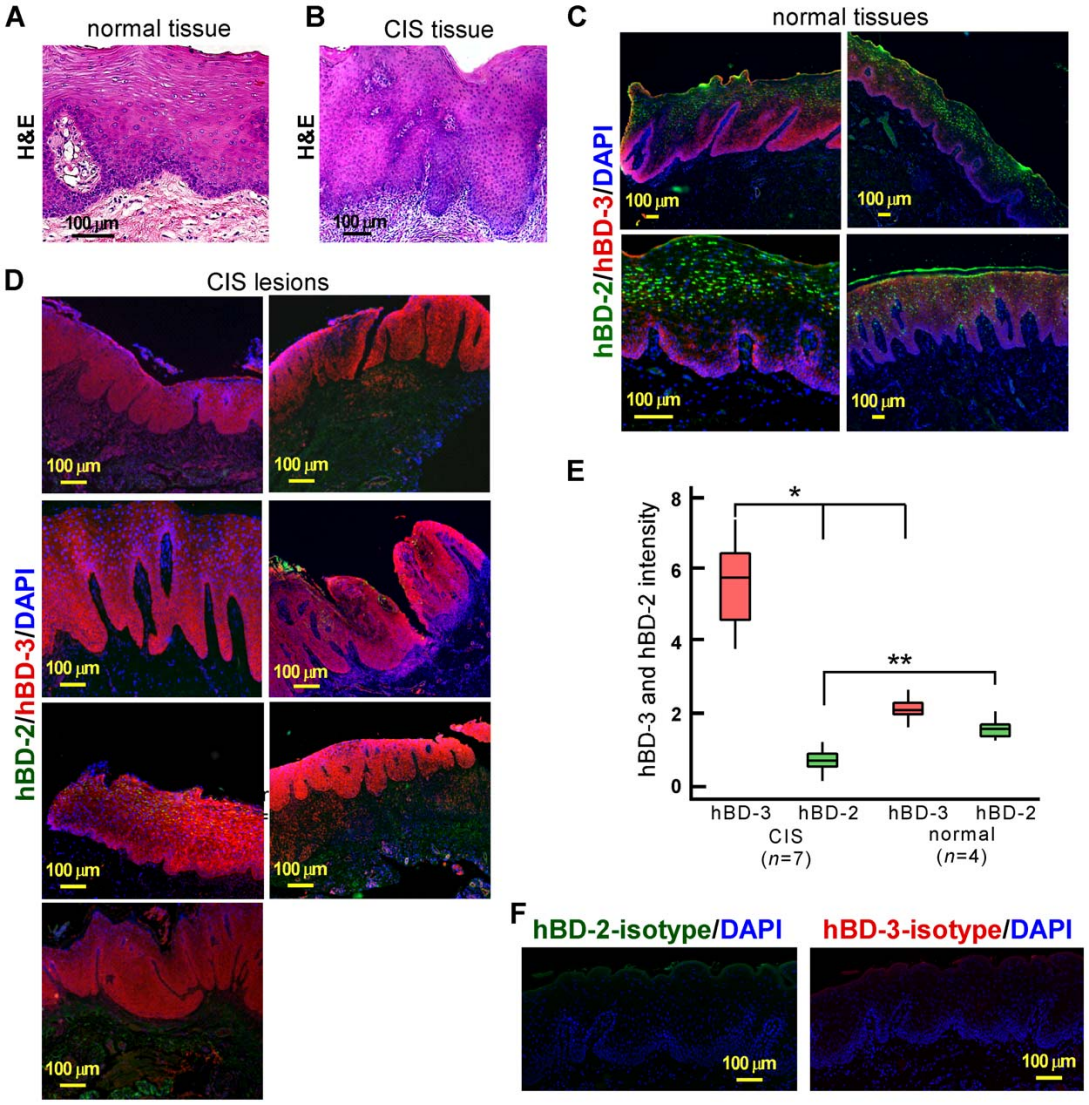
Expression of hBD-2 and hBD-3 in normal and carcinoma *in situ* (CIS) epithelia. (A and B) H&E images in normal oral epithelium (A) and CIS lesion (B) biopsy samples. (C and D) Immunofluorescent images of hBD-2 (green) and hBD-3 (red) in normal (C) and CIS (D) oral epithelial biopsies. Nuclei, blue (DAPI). (E) Quantification of immunofluorescence intensities of hBD-2 and hBD-3 over that of nuclei in normal oral epithelia and CIS biopsies. Epithelial biopsies were derived from 4 normal (*n* = 4) and 7 CIS (*n* = 7) individuals. The line drawn through the boxplot graph (E) represents the mean of the results and the line extending vertically from the box indicates the lowest and highest value in the data set. *, *p* = 0.00, ** *p* = 0.00. In normal oral epithelia, the mean of hBD-2 and hBD-3 was 1.53 and 2.11, respectively. In CIS tissues, however, the mean of hBD-2 and hBD-3 was 0.70 and 5.63, respectively. (F) Isotype controls for hBD-2 (left) and hBD-3 (right) using anti-goat IgG and anti-rabbit IgG antibodies, respectively. Nuclei, blue (DAPI).

We previously demonstrated that tumor cells overwhelmingly express hBD-3, but not hBD-2, in the oral CIS lesion [19]. To confirm the observation, additional normal (Figure 1A; one representative sample is shown in hematoxylin and eosin [H&E] staining) and CIS biopsies (Figure 1B; one representative is shown in H&E staining) were used for co-immunofluorescence staining using antibodies to hBD-2 and hBD-3. In normal oral epithelia, hBD-3 expression was detected only in the basal layers, while hBD-2 production could be observed in the differentiated superficial layers (Figure 1C; four samples are shown). However, in CIS, hBD-3 expression was observed throughout the lesion site in all biopsy samples, while hBD-2 expression was absent (Figure 1D; seven samples are shown; compare Figure 1C with Figure 1D). Immunofluorescence staining with the isotype control antibody to hBD-2 or hBD-3 did not detect any signals (Figure 1F). To compare the expression of hBDs between normal oral epithelia and CIS lesions quantitatively, immunofluorescence intensities of hBD-2 and hBD-3 were measured and normalized with that of nuclei in the epithelia of CIS and normal samples. The expression of hBD-3 was ∼3.7-fold higher in CIS samples (*n* = 7) compared with that in normal epithelia (*n* = 4), while hBD-2 expression was significantly higher in normal oral epithelia than CIS biopsies (Figure 1E). These results, in conjunction with those of Kesting et al [20], further support our original observation of the presence of an hBD-3-rich tumor microenvironment in CIS lesions.

**Figure 2:**
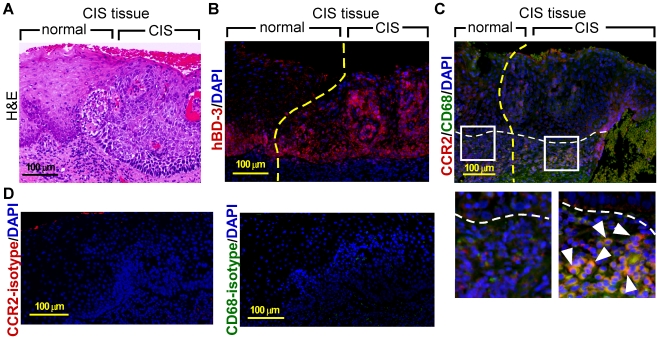
Localization of CCR2+/CD68+ macrophages in the CIS lesion. (A) H&E image of a CIS biopsy specimen. The CIS lesion and the adjacent normal region are demarcated. (B) Immunofluorescent staining of hBD-3 (red) in the consecutive section derived from (A). Dashed yellow line, boundary separating the CIS and adjacent normal region; nuclei, blue (DAPI). (C) Co-immunofluorescent image of CCR2 (red) and CD68 (green) in a consecutive section of (B). Several CCR2+/CD68 positive cells are indicated by white arrowheads (enlarged inset on the right) in the CIS lesion site. Dashed yellow line, boundary separating the CIS and adjacent normal region; dashed white line, basement membrane; nuclei, blue (DAPI). (D) Isotype controls of CCR2 (left panel) and CD68 (right panel) using sections derived from the same block of (B). Nuclei, blue (DAPI).

We have shown the association of hBD-3 with recruitment and infiltration of macrophages, but not CD3+ lymphocytes, into the tumor site [19]. Since macrophages express the chemokine receptor CCR2 for migration, we decided to determine whether hBD-3 expression was associated with recruitment of CD68+/CCR2+ cells. Figure 2 shows the expression of hBD-3 in the CIS lesion (Figure 2A and 2B) and co-stained CD68 and CCR2 accumulating in the CIS site, but not in the apparently normal region adjacent to the lesion (Figure 2C; the enlarged inset on the left, normal region adjacent to the CIS; on the right, CIS lesion, white arrowheads indicate CCF2+ macrophages), suggesting the association of the hBD-3-rich tumor microenvironment with recruitment of CCR2+ macrophages.

### Dissociation of expression of MCP-1 and tumor recruitment of macrophages

**Figure 3:**
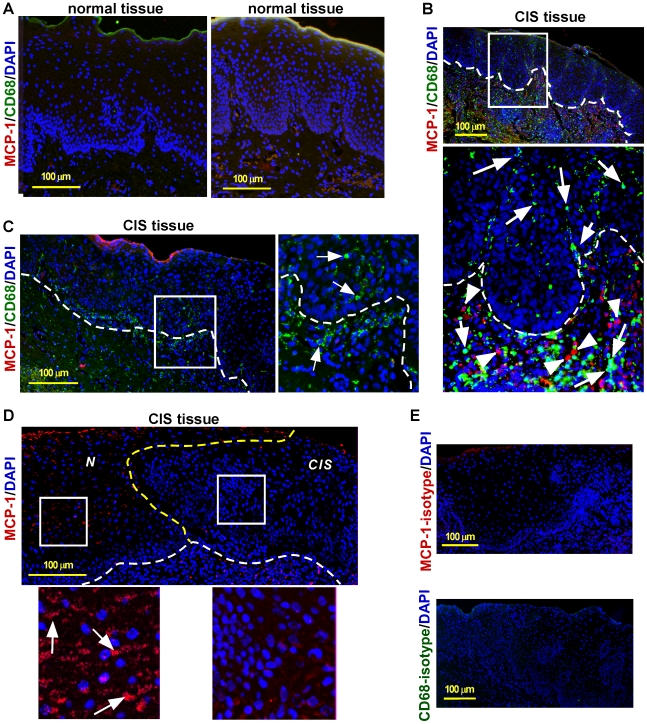
Localization of macrophages and MCP-1 expression in normal and CIS biopsy specimens. (A) Double-immunofluorescence of CD68 (green) and MCP-1 (red) in two normal oral epithelial biopsies. Nuclei, blue (DAPI). (B) CD68 (green) and MCP-1 (red) in a CIS biopsy section. Arrows, macrophages; arrowheads, MCP-1 expressing cells; dashed white line, basement membrane; nuclei, blue (DAPI). (C) CD68 (green) and MCP-1 (red) in a CIS biopsy section derived from a second patient. MCP-1 is undetectable in the entire section. Arrows in enlarged inset, macrophages; dashed white line, the basement membrane; nuclei, blue (DAPI). (D) Immunofluorescence of MCP-1 (red) in the CIS section (obtained from the same block in Figure 2B) derived from a third patient. MCP-1 expressing cells (enlarged inset on the left, white arrows) are detected in the normal region adjacent to the CIS site, but not in the CIS lesion. Dashed white line, basement membrane; dashed yellow line, boundary separating the CIS (*CIS*) and adjacent normal region (*N*); nuclei, blue (DAPI). (E) Isotype control for MCP-1 (upper panel) and CD68 (lower panel), respectively. Nuclei, blue (DAPI).

Since MCP-1 has been shown to be produced by tumor cells and correlated with recruitment and infiltration of immune cells in a variety of cancers [30], we investigated expression of MCP-1 and macrophage infiltration, using the macrophage cell surface marker CD68, to determine if this linkage is upheld in oral CIS. MCP-1 was either undetectable or sporadically expressed in the epithelium and lamina propria of normal oral tissue biopsy samples (Figure 3A) and no macrophages were detected (Figure 3A; two samples are shown). In clinically diagnosed CIS samples, however, macrophages were recruited abundantly to and infiltrated into the lesion site (Figure 3B and 3C). Interestingly, patterns of MCP-1 expression in these CIS samples were not consistent, nor correlated with recruitment and infiltration of macrophages (Figure 3B–D). In the CIS sample shown in Figure 3B, MCP-1 producing cells were located in the lamina propria adjacent to the basement membrane of the CIS lesion, while recruited macrophages were present in the area and infiltrated into the lesion site, where MCP-1 was not expressed (Figure 3B, enlarged inset; white arrows above the lamina propria). The CIS biopsy from a different patient showed no MCP-1 expression either in the lamina propria or in the CIS site, while intratumoral macrophages were evident (Figure 3C, enlarged inset). In another CIS sample, however, MCP-1 expression was observed in the normal region adjacent to the CIS, but not in the lesion (Figure 3D; enlarged inset on the left, normal region adjacent to the CIS lesion, MCP-1 expression is indicated with white arrows; enlarged inset on the right, the CIS site). Staining of the consecutive section shown in Figure 3D with antibodies to CCR2 and CD68 indicated the accumulation of CCR2+ macrophages in the CIS lesion (Figure 2C, enlarged inset on the right). Notably, in all seven CIS samples tested, tumor cells did not produce MCP-1 (Figure 3B–3D, enlarged insets). These results suggest that the expression of MCP-1 in CIS lesions varies and that there is no apparent correlation between MCP-1 expression and recruitment and infiltration of macrophages in oral tumors.

### Association of hBD-3 with tumorigenicity and tumor infiltration of host macrophages in nude mice

**Figure 4:**
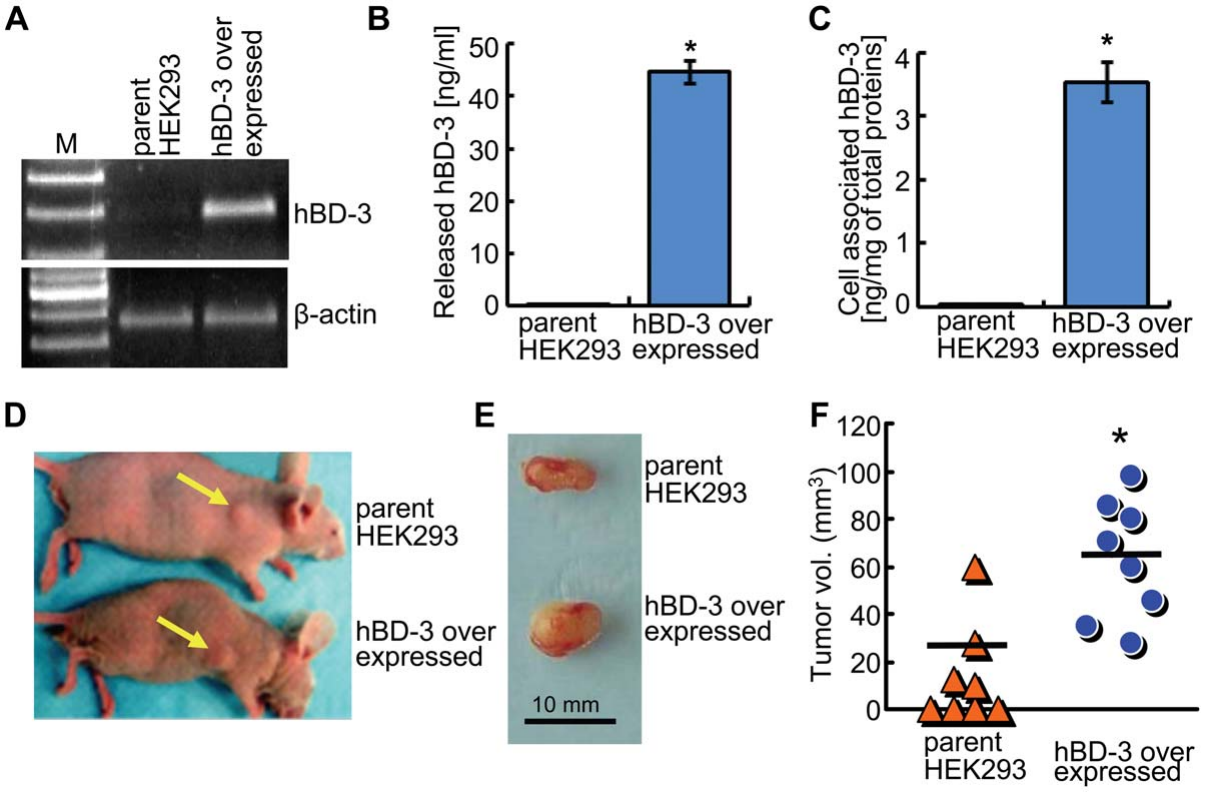
Xenograft tumors established with parent and hBD-3 overexpressing HEK293 cells in nude mice. (A) RT-PCR of hBD-3 on total RNA samples extracted from parent HEK293 and hBD-3 overexpressed HEK293 cells, respectively. (B and C) ELISA of hBD-3 using culture supernatants (B) or cell lysates (C) derived from parent HEK293 and hBD-3 overexpressed cells. HEK293 and hBD-3 overexpressing cells were cultured in serum-free medium for 3 days, followed by ELISA of collected media and cell lysates, respectively. *, *p* = 0.00. (D) Representative mice bearing tumors after 10 days post inoculation. Yellow arrows, inoculation sites. (E) Representative tumors isolated from mice inoculated with parent HEK293 cells and hBD-3 overexpressing cells. (F) The incidence and sizes of xenograft tumors generated using parent HEK293 and hBD-3 overexpressed HEK293 cells. The mean volume for each group of tumors is represented as black lines; in HEK293 tumors, the value is 27.9 mm^3^, while in hBD-3 overexpressed tumors, the value is 66.9 mm^3^. *, *p*<0.05.

To examine the *in vivo* role of hBD-3 in macrophage trafficking, we inoculated nude mice subcutaneously with tumorigenic human embryonic kidney 293 cells (HEK293) with or without hBD-3 overexpression. The transcription of hBD-3 in parent and hBD-3 overexpressing HEK293 cells was determined by RT-PCR, while secreted and cell-associated hBD-3 peptide in these cells were measured by ELISA analysis. The expression of hBD-3 mRNA was undetectable in parent HEK293 cells, while clearly detectable in the engineered hBD-3 overexpressing cell line (Figure 4A). The production of hBD-3 peptide in culture medium and the cell lysate was significantly higher in HEK293 cells that overexpressed hBD-3 compared with parent HEK293 cells (Figure 4B and Figure 4C), indicating that parent HEK293 cells do not express hBD-3. Ten days after inoculation, xenograft tumors formed in mice inoculated with parent HEK293 or hBD-3 overexpressing cells. However, hBD-3 overexpressing cells generated larger tumors than those established by parent cells (Figure 4D and 4E). In addition, hBD-3 overexpressing cells formed tumors in all eight inoculated sites, while only four tumors were established in nude mice injected with parent HEK293 cells (Figure 4F). The mean volumes for hBD-3 overexpressing tumors were about 66.9 mm^3^, while the mean parent HEK293 tumor volume was 27.9 mm^3^ (Figure 4F). These results suggest that hBD-3 overexpression increases the incidence of xenograft tumor formation and rate of tumor growth in nude mice.

**Figure 5:**
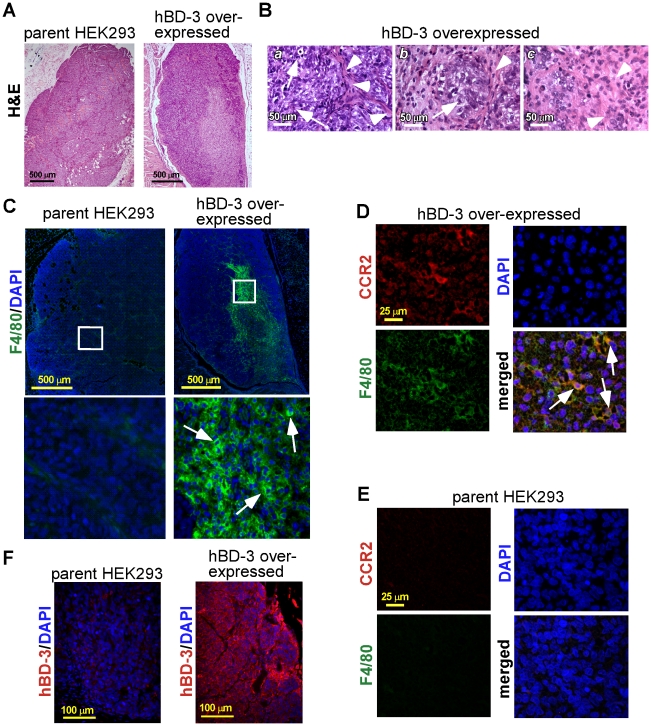
Characterizations of xenograft tumors in nude mice inoculated with parent and hBD-3 overexpressing tumorigenic cells. (A) H&E staining of xenograft tumor sections derived from parent and hBD-3 overexpressing HEK293 cells. (B) Histological features of the xenograft tumor established from hBD-3 overexpressing HEK293 cells. (*a*) cells under mitosis are indicated with arrows. Arrowheads indicate fibrous septae, (*b*) fibrous septae (arrowhead) divide the nodule into lobules of cell clusters arranged in an organoid pattern (arrow), (*c*) possible necrotic regions are indicated with arrowheads. (C) F4/80 (green) images of mouse macrophages in tumor sections derived from parent and hBD-3 overexpressing cells. Several F4/80+ cells are indicated by arrows in the hBD-3 overexpressed section (enlarged inset). Nuclei, blue (DAPI). (D) Double-immunofluorescent staining of F4/80 (green) and mouse CCR2 (red) in the hBD-3 overexpressed xenograft tumor section. Arrows indicate cells that express both CCR2 and F4/80 in the merged panel. (E) Double-immunofluorescent staining of F4/80 (green) and mouse CCR2 (red) in the parent HEK293 tumor section. Nuclei, blue (DAPI). (F) HBD-3 (red) in xenograft tumors generated from parent HEK293 (left panel) and hBD-3 overexpressing cells (right panel). Nuclei, blue (DAPI). Representative images from 2 independent experiments are shown.

H&E staining of the formalin-fixed, paraffin-embedded sections of the xenografts revealed a lighter-staining region within the central area of the hBD-3 overexpressing tumor (Figure 5A, right panel). However, this was not observed in tumors established by parent HEK293 cells (Figure 5A, left panel). Histological analysis indicated that the hBD-3 overexpressing xenograft tumors featured non-encapsulated circumscribed tumor nodules that contained relatively large, ovoid nuclei, mitotic structures, and possible necrotic regions (Figure 5B). The lighter-staining region, therefore, suggests necrosis in hBD-3 overexpressing tumors. To determine whether hBD-3 chemoattracts host macrophages, xenograft tumor sections were stained with the monoclonal antibody to F4/80 antigen, a murine specific macrophage marker [31]. The results showed massive host macrophage infiltration in tumors generated with hBD-3 overexpressing cells, but not in tumors formed by parent cells (Figure 5C, enlarged inset). Interestingly, in hBD-3 overexpressing tumors, macrophages were recruited preferentially to the area where the necrotic features were evident, suggesting a correlation between macrophages trafficking and necrosis (compare Figure 5A, right panel and Figure 5C, upper right panel). Double-staining of the specimen with antibodies to F4/80 and mouse CCR2 indicated infiltration of CCR2+ mouse macrophages in the tumor established by hBD-3 overexpressing cells (Figure 5D, CCR2+ mouse macrophages are indicated with white arrows). However, these cells were absent in the tumor formed by parent HEK293 cells (Figure 5E). Clearly, hBD-3 was expressed at high levels in tumors established from the hBD-3 overexpressing cells (Figure 5F).

### Induction of cytokine expression by hBD-3 in macrophages

**Figure 6:**
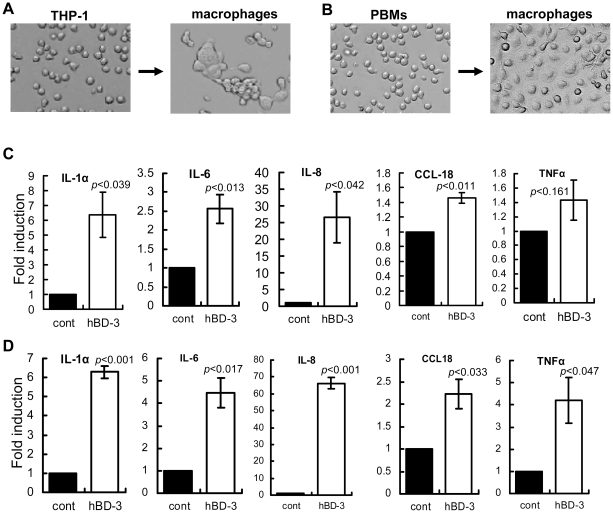
HBD-3 induced cytokines macrophages. (A and B) Macrophages differentiated from THP-1 cells using PMA stimulation (A) and from peripheral blood monocytes (PBMs) using macrophage-colony stimulating factor (M-CSF) treatment (B) [65]. Note macrophage-like morphological changes after differentiation. (C) Real-time quantitative RT-PCR of IL-1α, IL-6, IL-8, CCL18, and TNF-α in THP-1 cell-differentiated macrophages treated with hBD-3 (10 µg/ml) for 16 h. (D) Real-time quantitative RT-PCR of IL-1α, IL-6, IL-8, CCL18, and TNF-α in PBM-differentiated macrophages treated with hBD-3 (10 µg/ml) for 16 h. Experiments were repeated 3 times. *p* values are presented in each graph.

Tumor-derived factors in the tumor microenvironment can stimulate macrophages to produce a wide array of tumor-promoting molecules, such as chemokines, cytokines, and growth factors, to stimulate tumor cell proliferation, tumor angiogenesis and metastasis [12], [32]. To determine whether hBD-3 influences macrophages to produce tumor-promoting factors, we treated macrophages, which were differentiated from THP-1 monocytic cells *in vitro* using phorbol-myristate acetate (Figure 6A), with recombinant or synthetic hBD-3, followed by real-time quantitative RT-PCR (qPCR) analysis to assess mRNA levels of respective cytokines and chemokines. The results showed that hBD-3 induced the expression of IL-1α, IL-6, IL-8, and CCL18 (Figure 6C), suggesting that hBD-3 may activate macrophages and stimulate their tumor-promoting capacity. However, hBD-3 did not significantly induce TNFα expression in macrophages differentiated from THP-1 cells (Figure 6C). To determine whether human macrophages respond to hBD-3, we treated macrophages, which were differentiated from human peripheral blood monocytes (PBMs) *in vitro* with macrophage-colony stimulating factor (M-CSF) stimulation [33] (Figure 6B), with hBD-3 and subsequently extracted total RNA for qPCR analysis of the cytokine/chemokine transcripts. The results indicated that hBD-3 significantly induced expression of the cytokines/chemokines, including TNFα (Figure 6D). We also performed RT-PCR analysis using primers specific for IL-10, IL-17, and IL-23, cytokines that are involved in the tumor-related inflammation [12], [32]. However, the expression of these cytokines was not detected in macrophages with or without treatment (data not shown).

### Involvement of CCR2 in monocyte/macrophage migration in response to hBD-3

**Figure 7:**
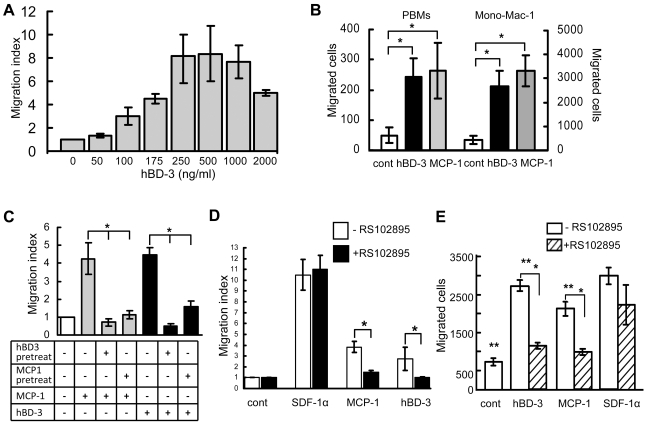
CCR2 mediated monocytic cell migration in response to hBD-3. (A) Dose-response of THP-1 cell migration in response to hBD-3. (B) Migration of PBMs and Mono-Mac-1 cells in response to hBD-3 (200 ng/ml) and MCP-1 (30 ng/ml). Migration of PBMs was determined by counting PBMs in 4 fields under a microscope in each lower chamber (Y-axis on left). To quantify Mono-Mac-1 migration, cells were collected from the lower chamber of transwell plates, centrifuged and suspended in PBS to count the total number of cells using a hemocytometer (Y-axis on right). cont, no chemoattractant control; *, *p*<0.05. (C) Effect of cross-desensitization on THP-1 monocytic cell migration in response to hBD-3 and MCP-1. Cells were desensitized by pretreatment with 10 µg/ml hBD-3 (hBD3 pretreat) or 100 ng/ml MCP-1 (MCP1 pretreat) for 1 h, followed by migration assays in response to MCP-1 (30 ng/ml) or hBD-3 (200 ng/ml). Results are representative of 3 independent experiments. *, *p*<0.05. (D and E) Effect of RS102895 on THP-1 (D) and Mono-Mac-1 (E) monocytic cell migration in response to hBD-3, MCP-1, and SDF-1α (control). Cells were pretreated with RS102895 at 20 µM for 2 h, followed by cell migration assays. hBD-3, 200 ng/ml; MCP-1, 30 ng/ml; SDF-1α, 50 ng/ml. cont, no chemoattractant control; * and **, *p*<0.05. THP-1 cell migration was calculated as migration indexes, while Mono-Mac-1 cell migration was quantified as the number of migrated cells.

We have previously shown that synthetic and recombinant hBD-3 induces migration of THP-1 cells comparably to MCP-1 [19]. In this report, we demonstrated that hBD-3 induced migration of THP-1 cells in a dose-dependent manner (Figure 7A). To further examine the chemotactic properties of hBD-3 to monocytic cells, we performed migration assays using human PBMs and the monocytic cell line Mono-Mac-1 in response to hBD-3. Migration data showed that hBD-3 induced cell migration of PBMs and Mono-Mac-1 cells (Figure 7B). Mono-Mac-1 was established from peripheral blood of a patient with monoblastic leukemia [34]. These cells retain distinct morphological, cytochemical, and immunological properties of monocytes [34]. Mono-Mac-1 cells express the chemokine receptor CCR2 and have been used to study monocytic functions *in vitro*, including chemotaxis [34], [35], [36]. CCR2 has been shown to play a nonredundant role as a major mediator of macrophage recruitment via MCP-1 [30], [37]. Because we have observed association of CCR2+ macrophage trafficking with tumor cell-produced hBD-3, but not MCP-1, in oral CIS lesions (Figure 2B, 2C; Figure 3B–D) and in xenograft tumors in nude mice (Figure 5C–E), we hypothesized that hBD-3 mediates monocyte/macrophage migration by acting through CCR2. Cross-desensitization of THP-1 monocytic cells by pretreatment with MCP-1 attenuated cell migration induced by hBD-3 and similarly, hBD-3 pretreatment blocked MCP-1 induced cell migration, suggesting that both hBD-3 and MCP-1 chemoattract monocytic cells through the same receptor, i.e., CCR2 (Figure 7C). To further confirm the importance of CCR2 in monocytic cell migration in response to hBD-3, we treated THP-1 and Mono-Mac-1 cells with the potent and selective CCR2 pharmacological inhibitor RS102895 [38], followed by *in vitro* migration assays in response to hBD-3, MCP-1, and SDF-1α, respectively. The CCR2 inhibitor blocked THP-1 and Mono-Mac-1 cell migration in response to hBD-3 and MCP-1, but not SDF-1α (Figure 7D and 7E). Collectively, these data indicate that hBD-3 induces monocyte/macrophage cell migration via CCR2.

## Discussion

### Role of hBD-3 in TAM trafficking

An oral CIS lesion is a histopathologic entity in which dysplastic cells, arising from the basal layer, occupy the full thickness of the epithelium from the basement membrane to the surface and in all likelihood progress to invasive carcinoma [39], [40]. We have previously demonstrated the spatiotemporal expression of hBDs at various stages of oral cancer and the possible role of hBD-3 in mediating the tumor-related inflammatory process [19]. In the present study, our results indicate that tumor cells within CIS lesions exclusively produce hBD-3, thereby generating the hBD-3-rich tumor microenvironment. The change in the expression pattern of hBDs between normal epithelium and the CIS lesion is probably related to the development and progression of oral cancer, since the hBD-3 expressing tumor cells are correlated with the accumulation of tumor-promoting TAMs in the lesion [19].

TAMs, derived from circulating monocytes, often make up a significant part of infiltrating immune cells in the tumor microenvironment and participate in the development and progression of tumors [30]. Clinical and experimental studies have shown that TAMs are frequently associated with poor prognosis in breast, prostate, bladder, and cervical cancers [2], [41], [42], [43], [44], [45]. Tumor and stromal cell produced chemokines, particularly MCP-1, have been associated with recruitment and infiltration of leukocytes to tumor sites [30]. Growth factors and cytokines are also described as chemotactic for monocytes/macrophages during tumor development [7] (Table 1). In oral squamous cell carcinomas, macrophage infiltration into and around cancer tissues is significantly correlated with tumor size, aggressiveness, invasion, and poor prognosis, while infiltrating T lymphocytes do not correlate with tumor progression [15], [16], [17]. However, tumor cell-derived molecules that recruit inflammatory cells to the oral cancer lesion are still largely undetermined [14], [18].

In the current report, we provide novel information about the role of hBD-3 in regulation of macrophage infiltration into tumors *in vivo*. Our data reveal that hBD-3 overexpressing cells, derived from the tumorigenic HEK293 cell line, form xenograft tumors in nude mice with massive infiltration of host macrophages compared with those generated from parent cells. HEK293 is an immortalized cell line established from the sheared adenovirus 5 DNA transformation of human embryonic kidney cells [46]. The cell line is tumorigenic and has been used as a tumor model for *in vitro* and *in vivo* assays of transformation, tumor progression, angiogenesis, and drug development [47], [48], [49], [50]. Gene expression profiles of the HEK 293 cell line documented by cDNA expression microarray analysis have identified low level expression of chemokines, cytokines, and growth factors under normal culture conditions [51], [52], [53], suggesting that HEK293 cell-derived molecules are unlikely to be involved in attracting host macrophages in nude mice. Thus, our findings indicate that hBD-3 is sufficient to induce migration of monocytes/macrophages *in vivo* and that hBD-3 exhibits a causal relationship with tumor infiltration of macrophages. It has been described that TAMs accumulate preferentially in the poorly vascularized, necrotic regions of tumors that are characterized by low oxygen tension [5]. Our results also revealed that accumulation of TAMs was particularly associated with necrotic regions of the xenograft tumors formed by hBD-3 overexpressing HEK293 cells.

### HBD-3 and tumorigenicity

Tumorigenicity of HEK293 cells is low when inoculated into nude mice and ectopic expression of oncogenic molecules, such as pituitary tumor transforming gene (PTTG), enhances oncogenic potential of transfected cells as shown by a higher incidence of tumor formation and rates of growth [54]. Our results showed that hBD-3 expressing HEK293 cells, but not parent cells, significantly increased the incidence and growth rates of xenograft tumors in nude mice, probably through infiltrating host macrophages. TAMs produce a wide array of tumor-promoting molecules, such as chemokines, cytokines, and growth factors, in the tumor microenvironment, to stimulate tumor cell proliferation, tumor angiogenesis, and metastasis [32]. For example, Lewis lung cancer (LLC) cells produce the extracellular matrix proteoglycan versican, which activates local macrophages to induce TNF-α secretion and subsequently stimulate LLC metastatic growth *in vivo*. This suggests that cancer cells can use components of the host innate immune system, such as TAMs, to generate a prevailing inflammatory microenvironment for metastasis [12]. In the current study, we demonstrate that hBD-3 can promote macrophage expression of IL-1α, IL-6, IL-8, and CCL18; i.e., cytokines and chemokines that are produced by TAMs as components of the cancer-related inflammation [5], [55]. HBD-3 also stimulates PBM-derived macrophages to produce TNFα. The proinflammatory and immunoregulatory cytokine IL-1 has been shown to play an important role in tumor angiogenesis [56]. Carmi et al. have demonstrated that macrophage-derived IL-1 activates infiltrating myeloid cells to produce VEGF, thus inducing endothelial cell migration, proliferation and organization into blood vessel-like structures and promoting tumor angiogenesis [57]. In their Matrigel plug system, Carmi and colleagues have shown that neutralization of IL-1 completely abrogated cell infiltration and angiogenesis and significantly reduced VEGF levels, thus inducing endothelial cell migration, proliferation and organization into blood vessel-like structures and promoting tumor angiogenesis [57]. IL-6 is a potent inflammatory cytokine that is considered a key tumor-promoting and antiapoptotic factor [58], [59]. IL-6 contributes to the induction of skin tumors [58], triggers malignant features in breast tumor mammospheres [60], and participates in suppression of antigen-specific anti-tumor immunity through up-regulation of macrophage B7-H4 expression [61]. TAM expression of IL-8 and a number of molecules, such as VEGF and TNF-α, have been implicated in enhanced angiogenesis [5], [62], while TAM-produced CCL18 can recruit naïve T cells to the microenvironment dominated by TAMs for possible T cell anergy [5]. Therefore, the tumorigenic effect of hBD-3 on xenograft tumor growth suggests that the inflammatory cells and molecules in the tumor microenvironment can affect a variety of transcriptional programs to promote tumor development and growth. Clearly, direct hBD-3 modulation of effector functions of TAMs *in vivo* needs to be further elucidated.

### CCR2 and monocytic cell migration in response to hBD-3

We and others have demonstrated that hBD-3 chemoattracts monocytes, including cells of monocytic cell lines and peripheral blood monocytes, *in vitro* [19], [28], [29]. The chemokine receptor CCR6 has been identified to mediate memory T cell and iDC migration in response to hBD-1 and hBD-2 [27]. HBD-3 has also been shown to be chemotactic for HEK293 cells overexpressing CCR6 [28]. However, the receptor that hBD-3 interacts with to recruit monocytic cells has not been identified, since these cells do not express CCR6 [28]. Here, we provide novel evidence that hBD-3 chemoattracts monocytic cells by acting through the chemokine receptor CCR2. Our cross-desensitization and the CCR2 inhibitor results indicate that hBD-3 and MCP-1 chemoattract monocytes by acting via the same CCR2 receptor. MCP-1 is a potent chemoattractant for monocytes, DCs, and natural killer (NK) cells [63]. MCP-1 interacts with the chemokine receptor CCR2 and triggers decoupling of G_i_-derived α subunit from Gβγ [64]. Activation of the CCR2 signaling pathway by MCP-1 initiates cascades of specific intracellular signaling events, including activation of phosphoinositide 3-kinases (PI3K) and phospholipase Cβ (PLCβ), intracellular calcium mobilization, and activation of PKC and ERK, resulting in cell migration [64]. RS102895 is a member of the spiropiperidine molecule class with potent and specific inhibitory properties to CCR2b, a splicing variant of the CCR2 gene that has higher binding affinity to MCP-1 and mediates chemoattraction and intracellular calcium influx by MCP-1 [36], [38], [64]. The binding of RS102895 to CCR2b blocks receptor binding of MCP-1, subsequently inhibiting intracellular calcium influx, cAMP inhibition, and chemotaxis by MCP-1 [38]. Our results suggest that hBD-3 may interact directly with CCR2 and subsequently activate its signaling pathways to induce monocyte migration. The notion is supported by recent work in which HEK293 cells that overexpress CCR2B migrate to the hBD-3:IgG fusion protein. In addition, mouse peritoneal exudate cells (PECs) derived from CCR2 deficient (*Ccr2* ^−/−^) C57BL/6 mice fail to migrate in response to hBD-3 (Joost Oppenheim; personal communication). Collectively, these results support our hypothesis that hBD-3 functions as a chemoattractant to recruit macrophages and that CCR2 plays a central role in mediating monocyte/macrophage migration in response to hBD-3.

In conclusion, we demonstrate herein that tumor cell-produced hBD-3 functions as a chemoattractant for recruitment of TAMs in the development of tumors and that hBD-3 chemoattracts monocytes/macrophages via the chemokine receptor CCR2. This novel mechanism is the first evidence of an hBD molecule orchestrating an *in vivo* outcome and demonstrates its importance in establishing a tumor-associated inflammatory microenvironment, which supports growth and progression of tumors.

## Materials and Methods

### Ethics statement

Tissue sample protocols for samples obtained from the Department of Oral Pathology, Case Western Reserve University School of Dental Medicine, and waiver of informed consent were approved by Case Cancer Institutional Review Board. All animal experiments were conducted in compliance with the Cleveland State University Institutional Animal Care and Use Committee. Written informed consents and protocols using human blood were approved by the Cleveland Clinic Institutional Review Board.

### Cell culture and reagents

THP-1 and HEK293 cells were obtained from American Type Culture Collection (Manassas, VA) and maintained in RPMI1640/10% FBS (Innovative Res., Novi, MI) and in DMEM/10% FBS, respectively. Mono-Mac-1 cells were provided by Dr. Sabina Sperandio (Centre de Recherche du CHUL, Canada) and cultured in RPMI1640/10% FBS. Differentiation of macrophages from THP-1 cells was performed as described by Tjiu et al [65]. Peripheral blood monocytes were prepared from human blood as previously described [66] and were differentiated to macrophages as described [33]. Recombinant hBD-3 was produced and tested for endotoxin contamination as we described previously [25]. Synthetic hBD-3 was purchased from Peptide International (Louisville, KY). SDF-1α and MCP-1 were purchased from PeproTech (Rocky Hill, NJ). Antibodies used in our studies were: goat polyclonal anti-hBD-2, goat polyclonal anti-MCP-1, mouse monoclonal anti-CCR2 (for human tissue), and rat monoclonal anti-F4/80 (Santa Cruz Biotech., Santa Cruz, CA); rabbit anti-mouse CCR2 (Abcam, Cambridge, MA); rabbit polyclonal anti-hBD-3 and mouse monoclonal anti-CD68 (Novus, Littleton, CO); AlexaFluor488-conjugated donkey antibodies to IgGs of mouse, goat, and rat as well as AlexFluor594-conjugated donkey antibodies to IgGs of rabbit and mouse (Invitrogen, Carlsbad, CA). Chromatographically purified IgGs of rabbit, mouse, goat, and rat were purchased from Invitrogen. Phorbol 12-myristate-13-acetate and RS10289 5 was purchased from Sigma-Aldrich (St Louis, MO) and dissolved in DMSO as stocks.

### Immunofluorescence microscopy

Formalin-fixed, paraffin-embedded biopsy specimens were obtained from the Department of Oral Pathology, Case Western Reserve University School of Dental Medicine. We previously described methods for immunofluorescence microscopy [19]. Briefly, each section (5 µm) was de-paraffinized in xylene and hydrated with serially diluted ethanol, followed by blocking with 10% donkey serum overnight at 4°C. After washing with PBS, each section was incubated with the respective primary antibody (1 h, room temperature), washed in PBS (3×10 min), and then stained with the compatible fluoresce dye-conjugated secondary antibody. For double immunofluorescence, consecutive staining by different primary and secondary antibodies was performed. Isotype controls were conducted using isotype-matched IgGs, corresponding to each primary antibody. Sections were mounted on slides with the VECTASHIELD Fluorescent Mounting Media (Vector Lab Inc., Burlingame, CA) containing DAPI to visualize nuclei. Immunofluorescent images were generated using a Leica DMI 6000B fluorescence microscope (Leica Microsystems, Bannockburn, IL) or an Olympus BX51 fluorescence microscope mounted with the Olympus DP71 camera (Olympus America Inc., Center Valley, PA). Immunofluorescence images were processed using the NIH ImageJ program [67]. To quantify expression levels of hBD-3, normal and CIS immunofluorescent images of hBD-2 and hBD-3 were acquired in 16-bit gray scale, respectively. Fluorescent densities on each of the antibody treated sections were measured with the NIH ImageJ program as described previously [19], [67]. The expression of hBD-2 and hBD-3 was represented as the ratio of relative fluorescence intensity of hBD-2 and hBD-3 over that of nuclei, respectively.

### Chemotaxis assay and ELISA for hBD-3

Chemotaxis assays were performed as described previously [19]. Briefly, serum-free RPMI1640 media containing hBD-3, MCP-1, or SDF-1α were added to each lower well of the Millicell-24 plate assemblies (5 µm membrane pore size) (Millipore, Billerica, MA). Cells (3×10^5^ in 100 µl of serum-free RPMI1640) were added to the upper wells. After incubation for 6 h, cells migrating into the lower-chamber were counted in 4 fields under a microscope (for PBMs), or collected and counted using a hemocytometer (for Mono-Mac-1 cells). Each experiment was repeated at least three times. Chemotactic activity was measured as either migration index, i.e., the ratio of the number of migrating cells in the lower well towards a chemoattractant when compared to medium alone, or the number of cells in the lower wells. The results were presented as mean ± SD of triplicate wells.

Concentrations of hBD-3 in cell lysates and medium supernatants were quantified using an enzyme-linked immunosorbent assay (ELISA) method as we described previously [68]. Briefly, 96-well immunoplates (R&D Systems, MN) were coated with 100 µl anti-hBD-3 antibodies (PeproTech) diluted to 1 µg/ml overnight at 4°C, followed by blocking with 1% bovine serum albumin (BSA) in phosphate buffered saline (PBS). Cell lysates, medium supernatants, and recombinant hBD-2 standards were incubated at room temperature for 1 h. The wells were washed 3 times with PBS containing 0.1% Tween 20 and incubated at room temperature with 100 µl of secondary antibody (PeproTech) diluted to 0.2 µg/ml, for 30 min. Each plate was washed 3 times and filled with 50 µl/well streptavidin-peroxidase (Roche Diagnostics; 1∶10,000 in PBS containing 0.1% Tween 20). Each plate was then incubated at room temperature for an additional 30 min, washed 3 times as described above, and incubated with 2,2-azino-bis-3-ethylbenzthiazoline-6-sulfonic acid (Roche Diagnostics, Branchburg, NJ) in the dark at room temperature for 20 min. Absorbance was measured at 415 nm with a microplate reader (Model 680, Bio-Rad, Hercules, CA).

### RT-PCR, and real-time quantitative RT-PCR

Total RNA was extracted using GeneElute mammalian total RNA isolation kit (Sigma, St. Louise, MO) following the manufacture's protocol as described previously [69]. Briefly, cells grown in 6-well plates were lysed using the lysis Buffer and the cellular lysates were centrifuged through the shredding columns. After collection of total RNA with the RNA column, the column was washed and the RNA was eluted using RNase-free H_2_O. Total RNA samples were quantified using a spectrophotometer at A_260_ and samples with the A_260_/A_280_ ratio ≥1.8 were used. For reverse transcription, RNA (1 µg) was used for the first strand cDNA synthesis using the SuperScript III reverse-transcriptase (Invitrogen) in a total volume of 20 µl according to the manufacturer's instructions. For RT-PCR analysis, the cDNA (2 µl) was used in a 25 µl of PCR amplification using *Tag* DNA polymerase (Invitrogen) with the following primers: IL-1α: 5′-CGCCAATGACTCAGAGGAAGA (forward) and 5′-AGGGCGTCATTCAGGATGAA (reverse), IL-6: 5′-TTCAATGAGGAGACTTGCCTG (forward) and 5′-ACAACAACAATCTGAGGTGCC (reverse), IL-8: 5′-GCCAGGAAGAAACCACCGGAAGGA (forward) and 5′-GGGGTCCAGACAGAGCTCTCTTCC, CCL18: 5′-CTCCTTGTCCTCGTCTGCAC (forward) and 5′-TCAGGCATTCAGCTTCAGGT (reverse), TNFα: 5′-CAGAGGGAAGAGTTCCCCAG (forward) and 5′-CCTTGGTCTGGTAGGAGACG (reverse), β-actin: 5′-GCTCGTCGTCGACAACGGCTC (forward) and 5′-CAAACATGATCTGGGTCATCTTCTC (reverse). For real-time quantitative RT-PCR (qPCR), total RNA (500 ng) was reverse transcribed using iScript cDNA synthesis kit (Bio-Rad) following the manufacture's protocol. Two µl of the reverse transcription (RT) reaction was used as a template for real-time PCR using a SYBR Green Supermix (Bio-Rad) with SYBE green 1 dye as the amplicon detector according to the manufacture's protocol. The gene for glyceraldehyde 3-phosphate dehydrogenase (GAPDHs was amplified as an endogenous reference. Amplification was performed at 40 cycles of 94°C for 15 s followed by 60°C for 1 min. Primers used for qPCR are listed in Table 2. Quantification was determined by using the comparative ΔΔC_T_ method as described by Peinequin et al [70]. Each qPCR was run in triplicates and the experiment was repeated at least 3 times.

**Table 2:** Primers used in real-time quantitative RT-PCR.

Gene names	Forward primer (5′→ 3′)	Reverse primer (5′→ 3′)	References
IL1-α	CGCCAATGACTCAGAGGAAGA	AGGGCGTCATTCAGGATGAA	[98]
IL-6	GGTACATCCTCGACGGCATCT	AGTGCCTCTTTGCTGCTTTCAC	[99]
IL-8	CTTGGCAGCCTTCCTGATTT	TTCTTTAGCACTCCTTGGCAAAA	[98]
CCL18	CTTTCCCCTTTCCCTTCAAC	GTGCTGAGCAAAACCATTCA	[100]
TNFα	TCTTCTCGAACCCCGAGTGA	CCTCTGATGGCACCACCAG	[98]
GAPDH	TCG GAG TCA ACG GAT TT	CCA CGA CGT ACT CAG C	[101]

### Transfection and mouse model

HBD-3 cDNA was cloned into pcDNA3.1 expression vector and the cDNA sequence was confirmed through DNA sequencing performed by the Genomics Core at Lerner Research Institute, Cleveland Clinic Foundation. Transfection of HEK293 cells was done with LipofectaminePlus following the manufacturer's protocol (Invitrogen). To generate xenograft tumors in nude mice (*nu*/*nu*, National Cancer Institute, Frederick, MD), hBD-3 overexpressing and parent HEK293 cells were trypsinized and suspended in PBS at 2×10^7^ cells/ml, respectively. Each animal was injected (2×10^6^ cells per injection) subcutaneously in two sites on opposite sides of the dorsum of the anterior part of the body. Ten days after inoculation, animals were sacrificed and tumor incidences were determined by counting tumors that could be identified visually. Isolated tumors were measured using a caliper and the tumor volumes were calculated with the equation *V* (mm^3^) = (*a*×*b* ^2^)/2, where *a* is the largest diameter and *b* is the perpendicular diameter in mm [71]. Three tumors from each group of mice were sectioned and subjected to immunofluorescence microscopy with the rat monoclonal antibody to F4/80, goat polyclonal antibody to mouse CCR2, and rabbit polyclonal antibody to hBD-3.

### Statistics

Results of migration to chemoattractants (migration index or the number of migrated cells) were compared with respective controls. The data were subjected to two-tailed paired Student's *t* test with two-sample equal variance for comparison of two groups. For quantification of immunofluorescence intensity, two-tailed paired Student's *t* test with two-sample unequal variance was used. *p*<0.05 was considered to be statistically significant. Data analyses were performed and graphs were generated using Minitab program (Minitab Inc.) and Exel 2003 (Microsoft, Seattle, WA).
